# Cardiovascular Health in a Single Community in Rural Haiti: A Cross-sectional Study

**DOI:** 10.48107/cmj.2021.07.01

**Published:** 2021-07-25

**Authors:** Vincenzo B. Polsinelli, Ketheline Rock Dantil, Vincent DeGennaro, Darius L. Fénelon, Dufens Pierre Louis, Joseph L. Izzo, Gene F. Kwan

**Affiliations:** 1 Department of Medicine, University of Pittsburgh Medical Center, Pittsburgh, Pennsylvania; 2 Friends of Fontaine, Fontaine, Haiti; 3 Innovating Health International, Port-au-Prince, Haiti; 4 Zanmi Lasante, Haiti; 5 Department of Medicine, Jacobs School of Medicine and Biomedical Sciences, Buffalo, New York; 6 Section of Cardiovascular Medicine, Boston University Medical Center, Boston, Massachusetts; 7 Department of Global Health and Social Medicine, Harvard Medical School, Boston, Massachusetts

**Keywords:** Haiti, Cardiovascular Health, Cardiovascular disease epidemiology, Hypertension

## Abstract

**Introduction:**

There is a growing burden of cardiovascular disease in low- and middle-income countries and assessment of cardiovascular health (CVH) may identify populations at risk for poor CVH.

**Methods:**

Between July 2014 and August 2014, we performed a household survey from a convenience sample among adult community members in rural northern Haiti. We used a modified World Health Organization STEPwise approach to chronic disease questionnaire to capture self-reported data on tobacco, diet, physical activity, and diabetes, and measured blood pressure and body mass index. We used an adapted American Heart Association definition and thresholds for determining ideal, intermediate, and poor cardiovascular health. We used linear and logistic regression to examine associations between socio-demographic characteristics with CVH score and ideal CVH.

**Results:**

Among 540 participants (mean [SD] age = 40.3 [17.1] years, 67% women), there was a high prevalence of poor CVH (n=476, 88.1%) compared with intermediate (n=56, 10.4%) and ideal (n=41, 7.6%) CVH. Ideal metrics for blood pressure (47%) and diet (26%) were least often met, while body weight (84%), physical activity (83%), and smoking (90%) were most often met. Men were associated with better CVH score (0.31, [0.04–0.59]; *P*=0.03), and being a farmer was associated with ideal CVH (*P*=0.006).

**Conclusion:**

In this community-based sample of a farming community in rural Haiti, very few adults had ideal CVH. Higher CVH score was associated with male sex, and farming as a primary occupation. Women and non-farmers may represent at-risk subgroups within this population. Blood pressure and diet may represent possible areas for improvement.

## INTRODUCTION

Haiti is the most impoverished nation in the Americas and has a high burden of cardiovascular disease (CVD) including hypertension, diabetes, and heart failure.^1–5^ In Haiti, ischemic heart disease and cerebrovascular disease are estimated to be the top two causes of death.^6^ One strategy to reduce CVD is primary prevention by improving cardiovascular health (CVH). CVD incidence and mortality is lower when a set of health behaviors (smoking, healthy diet, body mass index [BMI], and physical activity) and health factors (blood pressure, cholesterol, and glucose) are at ideal levels.^7, 8^ Identifying areas of improvement in CVH may inform policymakers and guide interventions to promote CVH through screening and public health initiatives. The Haiti Mortality, Morbidity and Service Use Survey (EMMUS-VI 2016–2017) reported a high prevalence of CVD risk factors like hypertension and diabetes, with low health service utilization. Studies comparing CVH in low- and middle-income countries may have region specific needs to reduce CVD, for example although in much of the world men are at greater risk of ischemic heart disease than women, there is no sex difference observed in sub-Saharan Africa, underscoring the need for regional data.^9^ Therefore, obtaining data at a regional level is extremely important for public health officials to make appropriate guidelines and recommendations. The objective of this manuscript is to report the CVH of a community sample of rural Haitians to identify the prevalence of ideal CVH and subpopulations at greatest risk.

## METHODS

From July 2014 to August 2014, we obtained a convenience sample of 572 community-based participants at their homes in Fontaine, a town of approximately 10,000 people in northern Haiti. A private hospital and non-profit clinic is available approximately 8km away, and the primary economic activity is farming. Research assistants trained by one of the investigators (V. Polsinelli) conducted interviews in Haitian Creole, the locally spoken language. Every household within a 3–5 square kilometer radius of the town center was offered participation in the study. Subjects at least 18 years of age were selected through a non-random voluntary basis in each household, members of the household who were not present were re-visited at a later time. If household members were living and working away from home, they were not sampled.

We used a modified step 1 and 2 of WHO STEPwise approach to chronic disease questionnaire.^10^ Routine STEPwise questions were used to obtain information pertinent to CVH including daily fruit and vegetable intake, physical activity by Global Physical Activity Questionnaire, tobacco use, and self-reported diagnosis of hypertension, diabetes, and cardiovascular diseases. Anthropometric measurements were obtained in each participant’s home. Height was measured on a flat surface with the participant standing upright without shoes using a tape measure and clipboard. Weight was measured with electronic scales (Seca 803, Chino, CA) on a flat, hard surface; participants were permitted to wear light clothing but no shoes. Brachial oscillatory blood pressure and pulse were measured three times using an automated device (Omron BP785 10 Series, Lake Forest, Illinois) after the participant was sitting at rest for at least 5 minutes with arm, back, and feet supported. The mean of the three measurements was used. Scales and blood pressure devices were calibrated by the manufacturer but not by the study team. Blood glucose samples were not taken.

We adapted the AHA definition for ideal, intermediate, and poor CVH to categorize the study population.^11^ Definitions of each category are outlined in Table S1. Each of the six available metrics were allocated a score of 0 (poor), 1 (intermediate), or 2 (ideal) based on convention.^11^ Three categories, smoking, diet, and diabetes, were allocated a score of 0 or 2 because data on recent tobacco cessation and impaired fasting glucose were not collected, diabetes was self-reported. Ideal CVH was defined as full achievement of all factors (CVH score = 12 out of 12). Intermediate CVH is defined as a participant having at least one intermediate metric, and no poor metrics. Poor CVH is defined as having at least one poor metric.

To determine differences in CVH score between sexes, we used *X*^2^ for categorical variables, Student’s *t*-tests for continuous variables and Wilcox rank sum for non-parametric data. Univariate (model 1) and multivariate (model 2) linear regression were used to examine associations between demographic characteristics and CVH score, and ideal CVH. Model 1 is age adjusted linear regression of the independent covariates, and model 2 is age adjusted and adjusted for all other listed covariates. A two-sided *P* value < 0.05 defined statistical significance. All statistical analyses were performed using Stata version 12 (StataCorp, LLC, College Station, Texas, USA). The Comité National de Bioéthique, Haiti and the Health Sciences Institutional Review Board of the State University of New York at Buffalo approved the study. We obtained informed consent from all participants.

## RESULTS

Among the 572 participants interviewed, complete information for CVH determination was available in 540 participants. The number of individuals screened was not recorded, but participation in study was high (estimated >95% of those invited participated). Participants’ mean (SD) age was 40.2 (17.1) years, and 66% were women. Only a minority completed secondary school (11%). Men compared with women were more often farmers (116 [62%] vs. 34 [9%]; *P*<0.001) less likely homemakers (4 [2%] vs. 189 [51%]; *P*<0.001). Smoking was more common in men (30 [16%] vs. 30[8%], *P*=0.005). Mean ± SD daily fruit or vegetable servings was 3.8 ± 1.6 for men and 4.0 ± 2.0 for women (*P*=0.17). Men reported more weekly metabolic minutes (MET-minutes [IQR]) of physical activity than women (11340[4800 – 23030] vs 4320[960 – 12000]; *P*<0.0001). There was no difference in systolic blood pressure (mmHg) between men and women (123.1 ± 19.7 vs. 123.8 ± 27.1; *P*=0.76). However, we observed a lower diastolic BP in men compared to women (78.1 ± 12.4 vs. 82.2 ±15.4; *P*=0.001), lower BMI (20.4 ± 2.9 vs 22.1 ± 4.4; *P*<0.0001). Fewer men were obese (1.6% vs. 6.3%; *P*=0.015).

**Figure 1.**
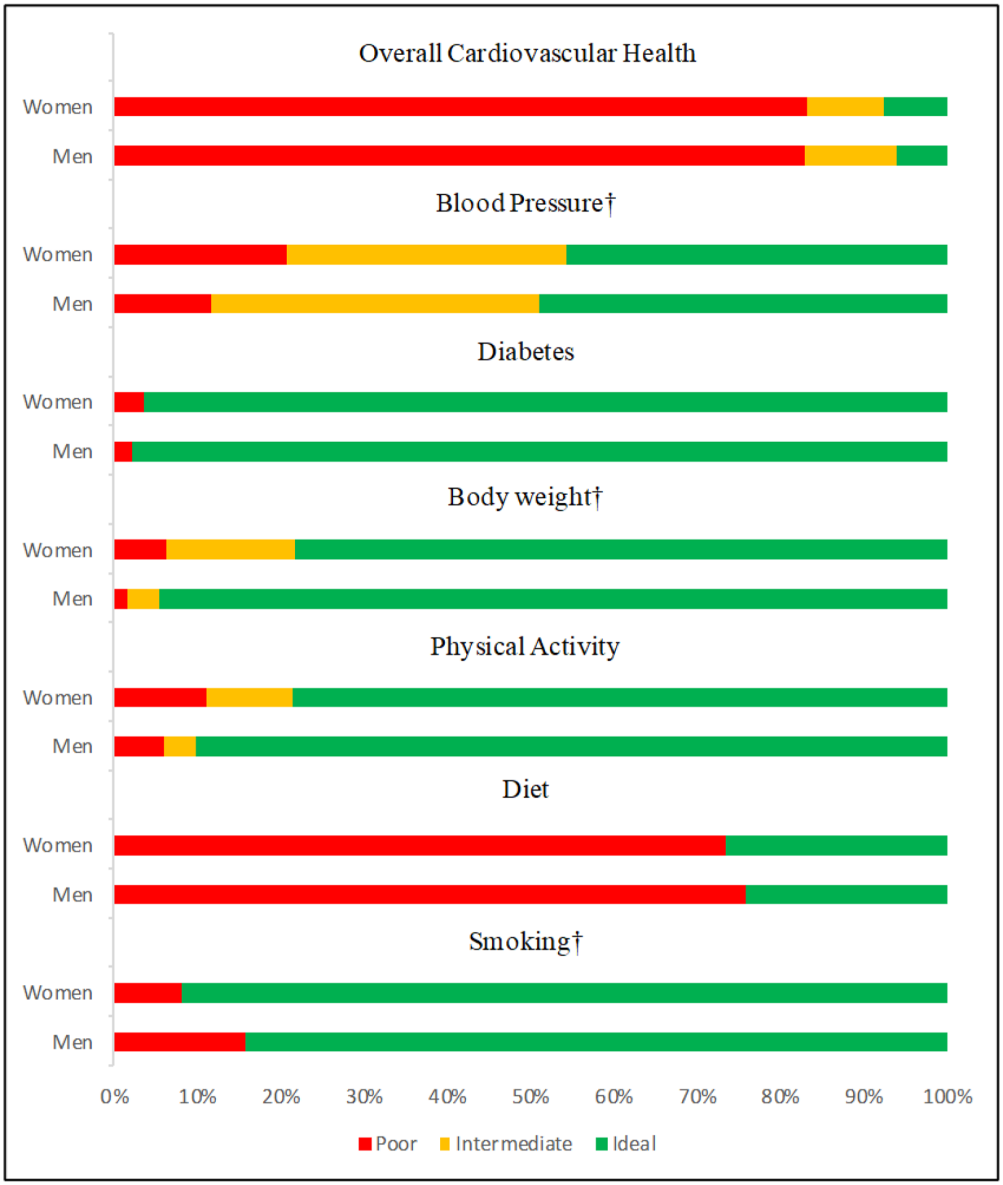
Distribution of cardiovascular health by sex

The prevalence of ideal, intermediate, and poor CVH was 7.2%, 9.8%, and 83.1% respectively and there were no differences by sex (*P*=0.6). Prevalence by each metric is shown in the Figure 1. Overall, ideal CVH metrics were more often observed among men compared to women. A greater proportion of men had ideal CVH metrics for blood pressure, body weight, and physical activity more often (*P*<0.02). One exception is smoking, as fewer women reported smoking tobacco compared to men (*P*=0.005). There were no differences for diabetes or diet.

Prevalence of ideal (green), intermediate (yellow), and poor (red) cardiovascular health metrics overall, and by category is separated by sex. Percentage of the total men or women is on the x-axis. Significant sex differences were observed in the categories of blood pressure, body weight, and smoking (P<0.05) †.

The mean ± SD CVH score was 9.1 ± 1.6 overall in the sample. Within the total cohort we observed a difference in CVH score by sex, 9.3 ± 1.5 among men and 9.0 ± 1.7 among women (*P* = 0.004). We evaluated the association between social and demographic determinants and total CVH score using multivariable regression—shown in the Table 1. After adjustments for age, the female sex was associated with worse CVH score. After adjustments for educational achievement and occupation, there were no observed associations with social and demographic characteristics. After adjustments for age, sex, and educational achievement, being a farmer was associated with ideal CVH (ß =2.38 (0.69 – 4.08; *P*=0.006).

## DISCUSSION

In this convenience sample of a rural agrarian community in northern Haiti, we described the distribution of CVH among Haitian adults, and several population characteristics associated with ideal CVH. Among all surveyed adults, we observed a very low prevalence of ideal CVH of 7.2%. Several ideal CVH metrics were more common among men compared to women, and an occupation of farmer was associated with ideal CVH. These data may help inform public health officials in rural Haiti to develop programs aimed at improvement of CVH.

Among our study participants, low smoking prevalence and smoking differences between men and women were similar to other studies in Haiti.^2, 6^ Comparing our data to a 2018 study from rural Haiti, the current study’s prevalence of overweight or obese was close to the reported estimate of 18%.^2^ Thus, these measurements of body weight appear consistent with rural lifestyle. Very few participants, (3% women; 2% men) had self-reported diabetes. This prevalence is likely an underestimate due to ascertainment bias as other studies utilizing fingerstick random glucose or hemoglobin A1c measurements have reported diabetes prevalence ranging from 5% to 20%.^2, 5, 6^ EMMUS-VI observed a relationship in diabetes prevalence and socioeconomic position. They observed diabetes prevalence to be lower for people in the lowest vs highest socioeconomic quintile for both women (11% vs. 18%) and men (5% vs. 10%), though the data from EMMUS-VI was based on an age range of 35 to 64, which is different than our studied sample. Other studies have observed high prevalence of food insecurity, and associations of food insecurity with illiteracy, poverty, less diverse diet, and death from cholera.^12, 13^ Thus, it is possible that regional food insecurity may explain our findings of lower BMI and lower prevalence of diabetes.

Our reported prevalence of ideal CVH of 7.2% is several-fold higher than other countries, including the United States (0.1%) and Ghana (0.3%), although we used a different scale in the current study.^7, 14, 15^ By each category, achievement of ideal CVH metrics was most often met within smoking, physical activity, diabetes, or BMI, and least often met within the hypertension and diet categories. These trends are similar to what has been observed in rural Ghana.^14^ Many lifestyle characteristics are optimized for ideal BP (low BMI, high level of physical activity); however, BP control levels are low.^1^ Thus improving access to medications to lower BP treatment may contribute to improved CVH in this population. Our findings of low ideal CVH are consistent with the high observed burden of heart failure and estimated burden of ischemic heart disease and stroke in Haiti.^4, 16, 17^

This study observed relevant trends with social and demographic characteristics. After adjustment for all other covariates, farmers were more likely to have ideal CVH; we speculate this is likely due to a physically active lifestyle necessary for farm work. Several metrics of CVH were observed to be worse in women, including our report of physical activity and weight. Culturally, women typically sell goods, or work around the home which are activities that may provide less physical activity and may explain why CVH metrics were worse in women. Furthermore, there exists in Haiti a high prevalence of pre-eclampsia which may explain poor CVH metrics, particularly BP, among women. These data suggest a need for CVH promotion particularly among women. Further investigation may be integrated at surveillance data including why women may be at higher risk, and linking those data with health system data to trial resource-effective ways to improve CVH.

This study has several limitations. Random sampling methods were not used but convenience sample chosen to optimize sample size, however, may biased towards participants who were wealthier and closer to the urban center. Re-sampling participants who were working during initial was inconsistently performed, and could bias the population toward less healthy individuals not able to work. Our diet metric was adapted as a simple and convenient measure, and the adaptation of the WHO STEPS instrument for development of the CVH score has not yet been validated. Diabetes was self-reported, however, we used a standardized instrument to minimize reporting bias and to facilitate comparisons to other populations.

## CONCLUSION

In conclusion, this study among this rural, Haitian population demonstrates a low prevalence of ideal CVH overall. Farmers are most likely to have ideal CVH, and women had worse CVH compared with men. We identified blood pressure and healthy diet as targets for interventions to improve CVH at a regional level. These data may have important implications for CVH health promotion and cardiovascular disease prevention and control.

## Supplementary Material

Supplement

## Figures and Tables

**Table 1.** Linear and logistic regression of covariates as predictors of CVH score and Ideal CVH. All covariates adjusted for age.

	*N (%)*	Model 1 Linear regression ß-Coeff. 95% CI (n=519)	Model 2 Linear regression ß-Coeff. 95% CI (n = 472)	Model 1 Logistic regression ß-Coeff. 95% CI (n=519)	Model 2 Logistic regression ß-Coeff. 95% CI (n = 467)
Covariate		CVH score		Ideal CVH	
**Sex**	519				
***Female***	350 (67.4%)	0.31* **(0.04 – 0.59)**	−0.03 (−0.42 – 0.35)	−0.15 (−0.86 – 0.56)	−0.95 (−1.96 – 0.06)
**Education**	472				
***No formal education***	156 (33.1%)	*Ref.*	*Ref.*	*Ref.*	*Ref.*
***Less than primary school***	120 (25.4%)	0.08 (−0.29 – 0.46)	0.04 (−0.35 – 0.43)	−0.56 (−1.52 – 0.41)	−0.35 (−1.36 – 0.65)
***Primary school***	144 (30.5%)	−0.05 (−0.44 – 0.35)	−0.10 (−0.52 – 0.31)	−1.28* **(−2.34 − −0.22)**	−0.78 (−1.89 – 0.34)
***Secondary school***	52 (11.0%)	0.61* **(0.09 – 1.13)**	0.53 (−0.03 – 1.08)	−0.50 (−1.65 – 0.65)	0.18 (−1.08 – 1.44)
**Occupation**	509				
***Employed***	68 (13.4%)	*Ref.*	*Ref.*	*Ref.*	*Ref.*
***Farmer***	124 (24.4%)	0.35 (−0.96 – 0.80)	0.43 (−0.09 – 0.94)	1.88* **(0.35 – 3.42)**	2.38* **(0.69 – 4.08)**
***Student***	72 (14.2%)	0.13 (−0.38 – 0.63)	0.15 (−0.38 – 0.68)	0.38 (−1.31 – 2.08)	0.62 (−1.13 – 2.37)
***Homemaker***	179 (35.2%)	−0.16 (−0.57 – 0.26)	−0.05 (−0.49 – 0.38)	1.06 (−0.46 – 2.59)	0.93 (−0.61 – 2.48)
***Retired***	66 (13.0%)	−0.31 (−0.82 – 0.19)	0.17 (−0.70 – 0.37)	1.33 (−3.48 – −0.22)	1.32 (−0.39 – 3.02)

* P < 0.05,

** P < 0.001,

Model 1: Individual covariates are adjusted for age and not with other covariates listed. Model 2: Covariates are adjusted for age and all other covariates listed (Sex, Education, Occupation).

**Table 2.** Definitions of cardiovascular health metrics, as adapted from the American Heart Association’s 2020 Strategic Impact Goals Committee.^11^

Overall CVH	≥ 1 poor metric	≥ 1 intermediate metric and 0 poor metrics	12 ideal metrics
**Smoking**	Active smoker	-	Non-smoker
**Physical Activity**	No Physical Activity	1–149 min/wk of moderate intensity, 1–74 min/wk of vigorous intensity, or 1–149 min/wk moderate plus vigorous intensity activity (whereby time in vigorous activity is doubled)	150 min/wk of moderate intensity, 75 min/wk of vigorous intensity, or 150 min/wk of moderate plus vigorous intensity activity (in which time in vigorous activity is doubled)
**Body Mass Index**	BMI ≥ 30 kg/m2	BMI 25–29.9 kg/m2	BMI <25 kg/m2
**Diabetes**	Self-reported diabetes	-	No self-reported diabetes
**Blood Pressure**	Treated BP >140/>90 and SBP ≥ 140 or DBP ≥ 90 mm Hg	SBP 120–139 or DBP 80–89 or treated BP <140/<90 mm Hg	BP <120/<80 mmHg
**Diet**	Diet score = 0	Diet score = 1	Diet score = 2–3

* Adaptations to previously published Cardiovascular Health score were made to Diet, smoking status, and diabetes due to incomplete data. Diet score (scale: 0 – 3) was calculated on the basis of one point for each of the following components, including; ≥ 4 servings of fruit or vegetables per day, ≥ 2 servings of fish per week, lowest tertile of reported daily sodium consumption. Intermediate smoking status was not obtained, diabetes status was limited to self-reporting.

## References

[1] Polsinelli VB, , Hypertension and aging in rural Haiti: results from a preliminary survey. Journal of Human Hypertension, 2017. 31(2): p. 138–144. 27465982 10.1038/jhh.2016.52

[2] DeGennaro V Jr., , Community-based diagnosis of non-communicable diseases and their risk factors in rural and urban Haiti: a cross-sectional prevalence study. BMJ Open, 2018. 8 (4): p. e020317. 10.1136/bmjopen-2017-020317 PMC5914767 29678978

[3] Pierce L, , Hypertension prevalence and knowledge assessment in rural Haiti. Ethnicity & Disease, 2014. 24(2): p. 213–9. 24804369

[4] Kwan GF, , Descriptive epidemiology and short-term outcomes of heart failure hospitalisation in rural Haiti. Heart, 2016. 102(2): p. 140–6. 26729609 10.1136/heartjnl-2015-308451 PMC4854668

[5] Jean-Baptiste ED, , Glucose intolerance and other cardiovascular risk factors in Haiti. Prevalence of Diabetes and Hypertension in Haiti (PREDIAH). Diabetes & Metabolism, 2006. 32(5 Pt 1): p. 443–51. 17110899 10.1016/s1262-3636(07)70302-6

[6] (MSPP), M.d.l.S.P.e.d.l.P., Haïti Enquête Mortalité, Morbidité et Utilisation des Services (EMMUS-VI 2016–2017). 2018, Institut Haïtien de l’Enfance: Pétion-Ville, Haïti.

[7] Folsom AR, , Community prevalence of ideal cardiovascular health, by the American Heart Association definition, and relationship with cardiovascular disease incidence. Journal of the American College Cardiology, 2011. 57(16): p. 1690–6. 10.1016/j.jacc.2010.11.041 PMC3093047 21492767

[8] Lloyd-Jones DM, , Defining and setting national goals for cardiovascular health promotion and disease reduction: the American Heart Association’s strategic Impact Goal through 2020 and beyond. Circulation, 2010. 121(4): p. 586–613. 20089546 10.1161/CIRCULATIONAHA.109.192703

[9] Sacco RL, , The Heart of 25 by 25: Achieving the Goal of Reducing Global and Regional Premature Deaths From Cardiovascular Diseases and Stroke: A Modeling Study From the American Heart Association and World Heart Federation. Circulation, 2016. 133(23): p. e674–90. 27162236 10.1161/CIR.0000000000000395

[10] Organization, W.H., WHO STEPS surveillance manual: the WHO STEPwise approach to chronic disease risk factor surveillance. 2005.

[11] Huffman MD, , Cardiovascular health behavior and health factor changes (1988–2008) and projections to 2020: results from the National Health and Nutrition Examination Surveys. Circulation, 2012. 125(21): p. 2595–602. 22547667 10.1161/CIRCULATIONAHA.111.070722 PMC3914399

[12] Richterman A, , Food insecurity and self-reported cholera in Haitian households: An analysis of the 2012 Demographic and Health Survey. PLoS Negl Trop Dis, 2019. 13(1): p. e0007134. 30699107 10.1371/journal.pntd.0007134 PMC6370226

[13] Rebick GW, , Food Insecurity, Dietary Diversity, and Body Mass Index of HIV-Infected Individuals on Antiretroviral Therapy in Rural Haiti. AIDS and Behavior, 2016. 20(5): p. 1116–22. 26350637 10.1007/s10461-015-1183-y PMC12862480

[14] van Nieuwenhuizen B, , Ideal cardiovascular health among Ghanaian populations in three European countries and rural and urban Ghana: the RODAM study. Internal and Emergency Medicine, 2018. 10.1007/s11739-018-1846-6 PMC6132772 29667109

[15] Benziger CP, , Low prevalence of ideal cardiovascular health in Peru. Heart, 2018. 10.1136/heartjnl-2017-312255 PMC6204974 29326111

[16] Network, G.B.o.D.C., Global Burden of Disease Study 2017 (GBD 2017) Haiti. 2018.

[17] Malebranche R, , Clinical and echocardiographic characteristics and outcomes in congestive heart failure at the Hospital of The State University of Haiti. American Heart Journal, 2016. 178: p. 151–60. 27502863 10.1016/j.ahj.2016.06.001

