# Supplementary material for: Cardiovascular Health in a Single Community in Rural Haiti: A Cross-sectional Study
Source: Caribb Med J. Author manuscript; Available in PMC 2021 Oct 6. (PMC8494238; doi:10.48107/cmj.2021.07.01)
Supplement: Supplement [file NIHMS1742427-supplement-Supplement.pdf]

Table S1. Definitions of cardiovascular health metrics, as adapted from the American Heart Association's 2020 Strategic Impact Goals Committee. [11]

**Overall CVH**	**≥ 1 poor metric**	**≥ 1 intermediate metric and 0 poor metrics**	12 ideal metrics
**Smoking**	Active smoker	-	Non-smoker
**Physical Activity**	No Physical Activity	1–149 min/wk of moderate intensity, 1–74 min/wk of vigorous intensity, or 1–149 min/wk moderate plus vigorous intensity activity (whereby time in vigorous activity is doubled)	150 min/wk of moderate intensity, 75 min/wk of vigorous intensity, or 150 min/wk of moderate plus vigorous intensity activity (in which time in vigorous activity is doubled)
**Body Mass Index**	BMI ≥ 30 kg/m2	BMI 25–29.9 kg/m2	BMI <25 kg/m2
**Diabetes**	Self-reported diabetes	-	No self-reported diabetes
**Blood Pressure**	Treated BP >140/ >90 and SBP ≥ 140 or DBP ≥ 90 mm Hg	SBP 120–139 or DBP 80–89 or treated BP <140/ <90 mm Hg	BP <120/<80 mmHg
**Diet**	Diet score = 0	Diet score = 1	Diet score = 2-3

* Adaptations to previously published Cardiovascular Health score were made to Diet, smoking status, and diabetes due to incomplete data. Diet score (scale: 0 – 3) was calculated on the basis of one point for each of the following components, including; ≥ 4 servings of fruit or vegetables per day, ≥ 2 servings of fish per week, lowest tertile of reported daily sodium consumption. Intermediate smoking status was not obtained, diabetes status was limited to self-reporting.
